# A machine learning model for the prediction of survival and tumor subtype in pancreatic ductal adenocarcinoma from preoperative diffusion-weighted imaging

**DOI:** 10.1186/s41747-019-0119-0

**Published:** 2019-10-17

**Authors:** Georgios Kaissis, Sebastian Ziegelmayer, Fabian Lohöfer, Hana Algül, Matthias Eiber, Wilko Weichert, Roland Schmid, Helmut Friess, Ernst Rummeny, Donna Ankerst, Jens Siveke, Rickmer Braren

**Affiliations:** 1 Department of Diagnostic and Interventional Radiology, Faculty of Medicine, Klinikum rechts der Isar, Technical University of Munich, Ismaninger Str. 22, DE-81675 Munich, Germany; 2 Department of Internal Medicine II, Faculty of Medicine, Technical University of Munich, Munich, Germany; 3 Department of Nuclear Medicine, Faculty of Medicine, Technical University of Munich, Munich, Germany; 4 Department of Pathology, Faculty of Medicine, Technical University of Munich, Munich, Germany; 5 Department of Surgery, Faculty of Medicine, Technical University of Munich, Munich, Germany; 6 Department of Mathematics, Technical University of Munich, Garching, Germany; 7 West German Cancer Center, University of Essen, Essen, Germany

**Keywords:** Machine learning, Diffusion magnetic resonance imaging, Pancreatic carcinoma, Radiomics, Survival analysis

## Abstract

**Background:**

To develop a supervised machine learning (ML) algorithm predicting above- *versus* below-median overall survival (OS) from diffusion-weighted imaging-derived radiomic features in patients with pancreatic ductal adenocarcinoma (PDAC).

**Methods:**

One hundred two patients with histopathologically proven PDAC were retrospectively assessed as training cohort, and 30 prospectively accrued and retrospectively enrolled patients served as independent validation cohort (IVC). Tumors were segmented on preoperative apparent diffusion coefficient (ADC) maps, and radiomic features were extracted. A random forest ML algorithm was fit to the training cohort and tested in the IVC. Histopathological subtype of tumor samples was assessed by immunohistochemistry in 21 IVC patients. Individual radiomic feature importance was evaluated by assessment of tree node Gini impurity decrease and recursive feature elimination. Fisher’s exact test, 95% confidence intervals (CI), and receiver operating characteristic area under the curve (ROC-AUC) were used.

**Results:**

The ML algorithm achieved 87% sensitivity (95% IC 67.3–92.7), 80% specificity (95% CI 74.0–86.7), and ROC-AUC 90% for the prediction of above- *versus* below-median OS in the IVC. Heterogeneity-related features were highly ranked by the model. Of the 21 patients with determined histopathological subtype, 8/9 patients predicted to experience below-median OS exhibited the quasi-mesenchymal subtype, whilst 11/12 patients predicted to experience above-median OS exhibited a non-quasi-mesenchymal subtype (*p* < 0.001).

**Conclusion:**

ML application to ADC radiomics allowed OS prediction with a high diagnostic accuracy in an IVC. The high overlap of clinically relevant histopathological subtypes with model predictions underlines the potential of quantitative imaging in PDAC pre-operative subtyping and prognosis.

**Electronic supplementary material:**

The online version of this article (10.1186/s41747-019-0119-0) contains supplementary material, which is available to authorized users.

## Key points

- Pancreatic cancer is a morphologically and genetically heterogeneous tumor entity.
- Histopathological subtypes of pancreatic cancer display different therapy response and survival.
- Whole-tumor radiomic analyses can capture and assess heterogeneity and its impact.
- This study applies machine learning to radiomic features derived from diffusion-weighted magnetic resonance imaging.
- The algorithm developed allowed the prediction of overall survival and tumor subtype with high diagnostic accuracy in an independent validation cohort.

## Background

Pancreatic ductal adenocarcinoma (PDAC) carries amongst the poorest prognoses of all cancers. Tumors exhibit heterogeneity on a genetic, transcriptomic, and proteomic level, which manifests itself in a complex tissue architecture including tumor cells, various fibroblast, and immune cell populations embedded in a poorly vascularized, dense stroma [1]. Despite its overall dismal prognosis, recent research has identified specific molecular subtypes with distinct therapy response and outcome. Amongst these, the so-called classical phenotype shows improved chemotherapy response and survival compared to the so-called quasi-mesenchymal or basal-like subtype underlining the urgent requirement for advanced techniques for precise pre-treatment patient stratification [2, 3]. This is crucial for adequate patient management, based on informed decision processes, clinical trial design, and outcome interpretation.

In heterogeneous tumors such as PDAC, biopsies carry a risk of tissue undersampling. In contrast, imaging inhabits a unique niche in precision medicine in that it can provide volumetric information non-invasively. Radiomics, the process of derivation of quantitative analytics from medical imaging data [4], represents a substantial advance over traditional image analysis workflows. In fact, it leverages data science and machine learning (ML) techniques to exploit non-intuitive image content and integrate it with clinical information to create a generalizable model capable of predicting biological features or the course of disease [5].

Since PDAC is a relatively rare tumor entity, typically only treated in specialized interdisciplinary centers, there is still a paucity of radiomic studies aiming to assess pertinent metrics such as patient survival or histopathological subtypes. Our aim was to apply a standardized, reproducible radiomic workflow to diffusion-weighted imaging (DWI)-derived apparent diffusion coefficient (ADC) maps, pipelined to a ML model capable of predicting overall survival and histopathological subtypes, trained and independently validated on two cohorts of PDAC patients.

## Methods

### Study design

Data collection, processing, and analysis were approved by the institutional ethics committee Ethikkommission der Medizinischen Fakultät der Technischen Universität München, protocol number 180/17S and 5573/12. The study was designed as a retrospective cohort study with a prospectively accrued, retrospectively enrolled independent validation cohort. The study endpoint was defined as overall survival. The requirement for written consent was waived for the retrospective cohort whilst written consent was obtained for the independent validation cohort for image acquisition and analysis of the imaging data. The two cohorts were accrued by the department of radiology (training cohort) and the clinic for nuclear medicine (independent validation cohort) at the same university hospital. All procedures were carried out in accordance with pertinent laws and regulations.

We considered patients with final histopathological diagnosis of PDAC of the head and body for inclusion in the study. Patients who did not have a final diagnosis of PDAC, had undergone treatment such as chemotherapy or resection prior to enrolment, refused treatment or study inclusion, died within the first 2 months of follow-up (to limit bias from postoperative complications), did not undergo the full imaging protocol, or did not have technically sufficient imaging available due to motion artefacts precluding imaging analysis were excluded. For inclusion in the training cohort, we retrospectively considered 206 consecutive patients, who presented at our institution between 2008 and 2013 and underwent imaging at the department of radiology with a suspected finding of PDAC. The median time between imaging and final histopathological diagnosis was 8 days (range 5–11). The follow-up interval was defined as 5 years post-imaging. Follow-up was handled by the departments of surgery and internal medicine. After exclusions, a total of 102 patients were included in the study as the training cohort.

Prospective patients were accrued from 2013 onwards as part of an effort to evaluate imaging performance and prognostic value of 3-T magnetic resonance imaging (MRI). Participants underwent 3-T MRI evaluation for suspected finding of PDAC. Of 62 consecutive patients who were considered for inclusion, 30 patients fulfilled the enrolment criteria and designated as the independent validation cohort*.* The median time between imaging and final histopathological diagnosis was 7 days (range 5–12).

Clinical data was sourced from the clinical information system. Radiomics data was generated during data analysis. For exclusion of bias, data analysis was performed in pesudonomyzed form and handled by separate individuals (G.K. and S.Z.). Data analysis was performed starting in June 2018. Patient flowcharts and the complete STROBE (*Strengthening the reporting of observational studies in epidemiology*) [6] checklist can be found in Additional file 1.

### Clinical variables

The following clinical data was collected for patients in the training and independent validation cohorts: age at diagnosis, sex, p/cTNM, resection status, grading, tumor volume in millilitres (as supplied in the final histopathological report), ECOG (Eastern Cooperative Oncology Group) performance status [7], and chemotherapy regimen. Where applicable and available, pre-operative CA19-9 levels and lymph-node ratios, *i.e.*, the ratio of the number of metastatic lymph nodes to the number of dissected lymph nodes, were noted. Overall survival was defined as the time from diagnosis to death.

### Imaging data acquisition

The 102 training cohort patients underwent MRI at 1.5T (Magnetom Avanto, release VB17, Siemens Healthineers, Erlangen, Germany). The protocol included the following sequences: axial and coronal T2-weighted (slice thickness, 5 mm); axial T1-weighted (slice thickness, 3 mm) before contrast injection (2 mL/kg body mass Gd-DTPA (Magnevist, Bayer HealthCare, Whippany, USA)) and during the arterial, pancreatic parenchymal, portal-venous, systemic venous, and delayed phases (as determined by testing bolus injection); axial unidirectional DWI at *b* values of 0, 50, 300, and 600 s/mm^2^ with echo-planar imaging readout and ADC map calculation. ADC map reconstructions had a spatial resolution of 5.5 × 5.5 × 5 mm (*x*, *y*, *z*) to a 192 × 192 matrix. Furthermore, single-shot T2-weighted magnetic resonance cholangiopancreatography was performed and reconstructed as a radial maximum intensity projection series. The independent validation cohort included 30 patients who underwent MRI on a 3-T clinical positron emission tomography MRI scanner (Biograph mMR, release VB18, Siemens Healthineers, Erlangen, Germany) at the nuclear medicine department. The protocol was performed as above with the following alterations: ADC-map reconstructions were 5.1 × 5.1 × 5.1 mm (*x*, *y*, *z*) to a 192 × 192 matrix; furthermore, an axial spectral adiabatic inversion recovery fat-suppressed post-contrast sequence at 5 mm and a whole-body positron emission tomography scan after application of ^18^F-fluorodeoxyglucose were included. The imaging protocols used and the technical hardware specifications of the MRI machines remained unaltered during the data acquisition period. Sequence parameters can be found in Table 1.

**Table 1:** Acquisition parameters for the training and independent validation cohorts

	Training cohort	Independent validation cohort
System	Siemens Magnetom Avanto	Siemens Biograph mMR
Software version	VB17	VB18
Anatomic sequences	Axial and coronal T2-weighted HASTE, 5-mm thickness	Axial and coronal T2-weighted HASTE, 5-mm thickness
	Axial T1-weighted VIBE, 3-mm thickness	Axial T1-weighted VIBE, 3-mm thickness
Dynamic study	Axial T1-weighted SPAIR	Axial T1-weighted T1 SPAIR
DWI acquisition	Axial low-resolution EPI, *b* = 0, 50, 300, 600 s/mm^2^	Axial low-resolution EPI, *b* = 0, 50, 300, 600 s/mm^2^
ADC fit	Linear, *b* = 50, 300, 600	Linear *b* = 50, 300, 600
Acquisition voxel size (*x*, *y*, *z*)	5.5 × 5.5 × 5.5 mm	5.1 × 5.1 × 5.1 mm
ADC reconstruction matrix	192 × 192	192 × 192
ADC field of view	360 × 360	360 × 360

*ADC* Apparent diffusion coefficient, *DWI* Diffusion-weighted imaging, *HASTE* Half-Fourier acquisition single-shot turbo spin-echo, *SPAIR* Spectral attenuated inversion recovery, *VIBE* Volume interpolated breath-hold examination

### Data segmentation

**Fig. 1:**
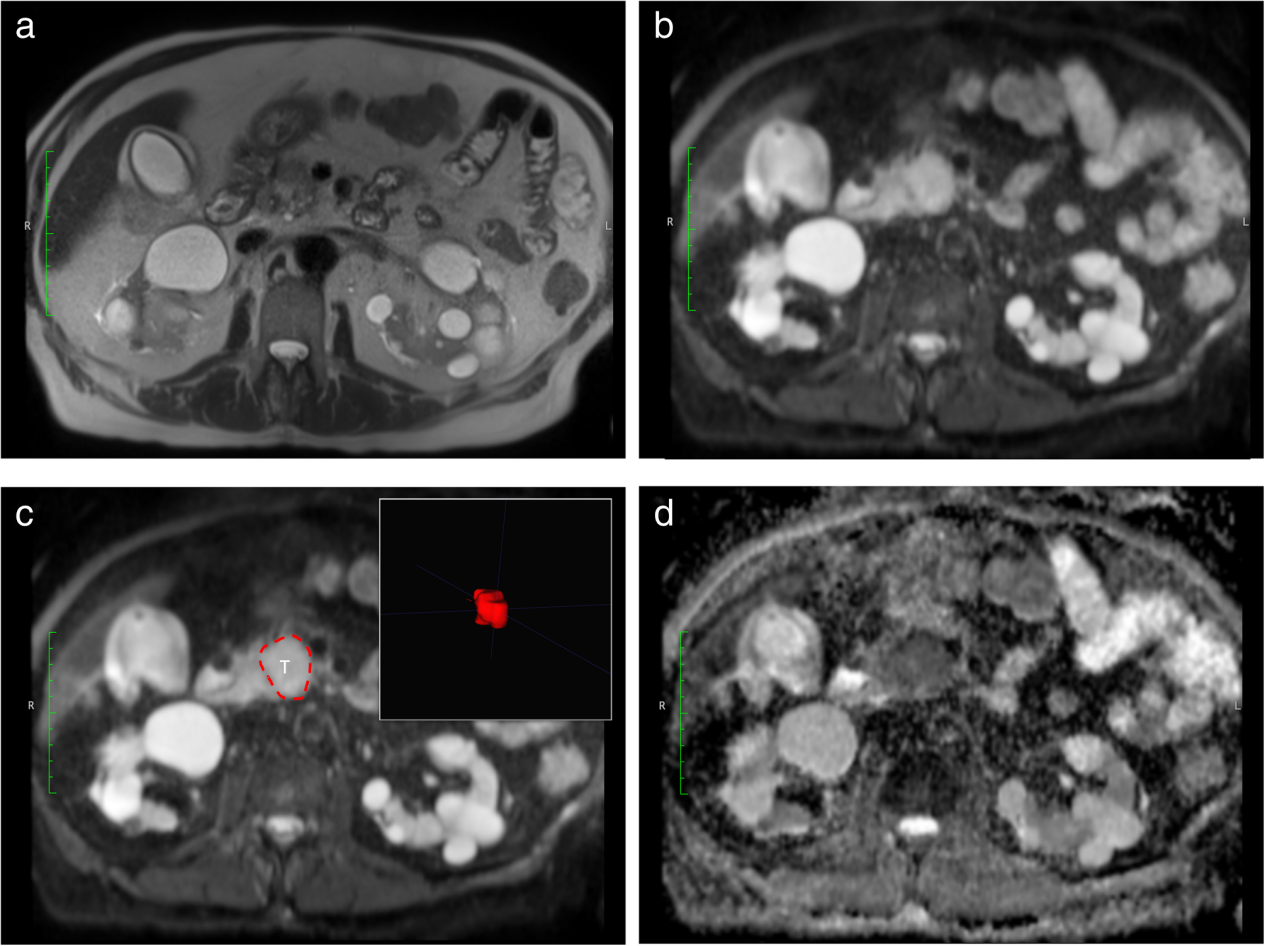
Exemplary case showing a ductal adenocarcinoma of the pancreatic head on T2-weighted images (**a**), *b* = 600 s/mm^2^ (**b**), the segmentation image including a three-dimensional rendering (inset) and a region-of interest (T) of the tumor (**c**), and the apparent diffusion coefficient map (**d**)

Pseudonomyzed datasets were exported from the hospital picture archiving system onto a radiological workstation and segmentation was performed under standardized lighting conditions by consensus reading of two experienced observers (G.K. and S.Z.) and quality-controlled by an abdominal radiologist with more than 10 years of experience in pancreatic MRI (RB segmentation was performed manually on the *b* = 600 s/mm^2^ images and transferred to the ADC maps). An exemplary case is shown in Fig. 1. All sequences were available to observers for anatomical correlation.

### Inferential statistical modelling

For assessing potential clinical confounding parameters introducing bias to the survival prediction, survival time was modelled in both cohorts using a multivariate Cox proportional hazards model. 95% confidence intervals were calculated by bootstrap resampling. The distributions of clinical variables were compared between groups using Fisher’s exact test. For subsequent ML modelling, the two cohorts were dichotomized by median overall survival to yield two sub-cohorts of equal size. Receiver operator characteristic (ROC) thresholds were evaluated with the Kolmogorov-Smirnov statistics. Biostatistical modelling was performed in SPSS version 25 (IBM, Armonk, USA). For all inferential statistical procedures, a *p* value lower than 0.05 was considered significant.

### Image postprocessing, radiomic feature extraction, and ML modelling

All steps of image postprocessing, feature extraction, feature preprocessing, feature engineering, and ML modelling are detailed in the Additional file 1. In brief, radiomic features were derived after intensity discretization to 32 bins using PyRadiomics version 2.1 [8] yielding a total of 1.688 features 19 first-order statistics, 16 three-dimensional shape-based, 10 two-dimensional shape-based, 24 gray-level co-occurrence matrix, 16 gray-level run-length matrix, 16 gray-level size zone matrix, 5 neighbouring gray tone difference matrix, and 14 gray-level dependence matrix features as well as Laplacian of Gaussian-filtered, wavelet-decomposition-based (using the coiflet 1 function), square, exponential, gradient, square-root, logarithm, and local binary-pattern filtered versions of these features. Feature preprocessing was applied to eliminate non-reproducible and unstable features, leading to the exclusion of 1184 features as detailed in Additional file 1, section 2 (Feature preprocessing) and previously described in [8]. In brief, features with segmentation-resegmentation instability, features unstable in reference tissues between the two MRI systems, and features yielding constant, nil, or missing values were removed. In total, 504 features were retained. Feature values were normalized to the (0,1) interval. A random forest [9] classifier was fit in a supervised fashion with survival above *versus* below median serving as label to the training cohort radiomic features. Hyperparameter tuning and algorithm development were performed by tenfold nested cross-validation on the training set with an internal loop used for automated hyperparameter optimization by randomized grid searching and the independent loop used for algorithm validation. Feature importance was assessed by the inbuilt feature importance metrics of the algorithm based on the decrease of node Gini impurity (a metric of misclassification rate and thus of the quality of the split at each decision tree node, compare Section 9.2.3, *Classification Trees* as explained by Hastie et al [10] and by recursive feature elimination. The algorithm was then tested for predictive sensitivity, specificity, and ROC area under the curve (ROC-AUC) in an independent validation cohort by using Fisher’s exact test on the contingency table of the correctly and incorrectly classified cases. To assess the prognostic significance of large-area low gray-level emphasis, the algorithm was refit to the training data using only this feature and tested on the independent validation cohort. All analyses were carried out using the Python programming language.

### Histopathological workup of tumor samples

Histopathological staining and immunohistochemical workup were performed as described by Muckenhuber et al. [11]. In brief, staining for the markers HNF1a and KRT81 was carried out and tumors categorized into three subtypes: classical, exocrine, and quasi-mesenchymal. Tumors not positive for either marker were designated as unclassifiable. Classical, exocrine, and unclassifiable tumors are onwards referred to as “non-quasi-mesenchymal”.

## Results

The distribution of clinical parameters did not differ significantly between the training and the independent validation cohorts. Amongst the clinical parameters, the choice of chemotherapy regimen (gemcitabine *versus* Folfirinox) was significantly associated with overall survival in the training cohort but not in the testing cohort, and the percentage of patients receiving each regimen was identical (with ~ 70% of patients receiving gemcitabine in each cohort (*p* = 1.000, Fisher’s exact test). Metastatic status at baseline was significantly associated with diminished survival in both cohorts and was also identically distributed (~ 25% of patients, *p* = 0.812, Fisher’s exact test) in both cohorts.

**Fig. 2:**
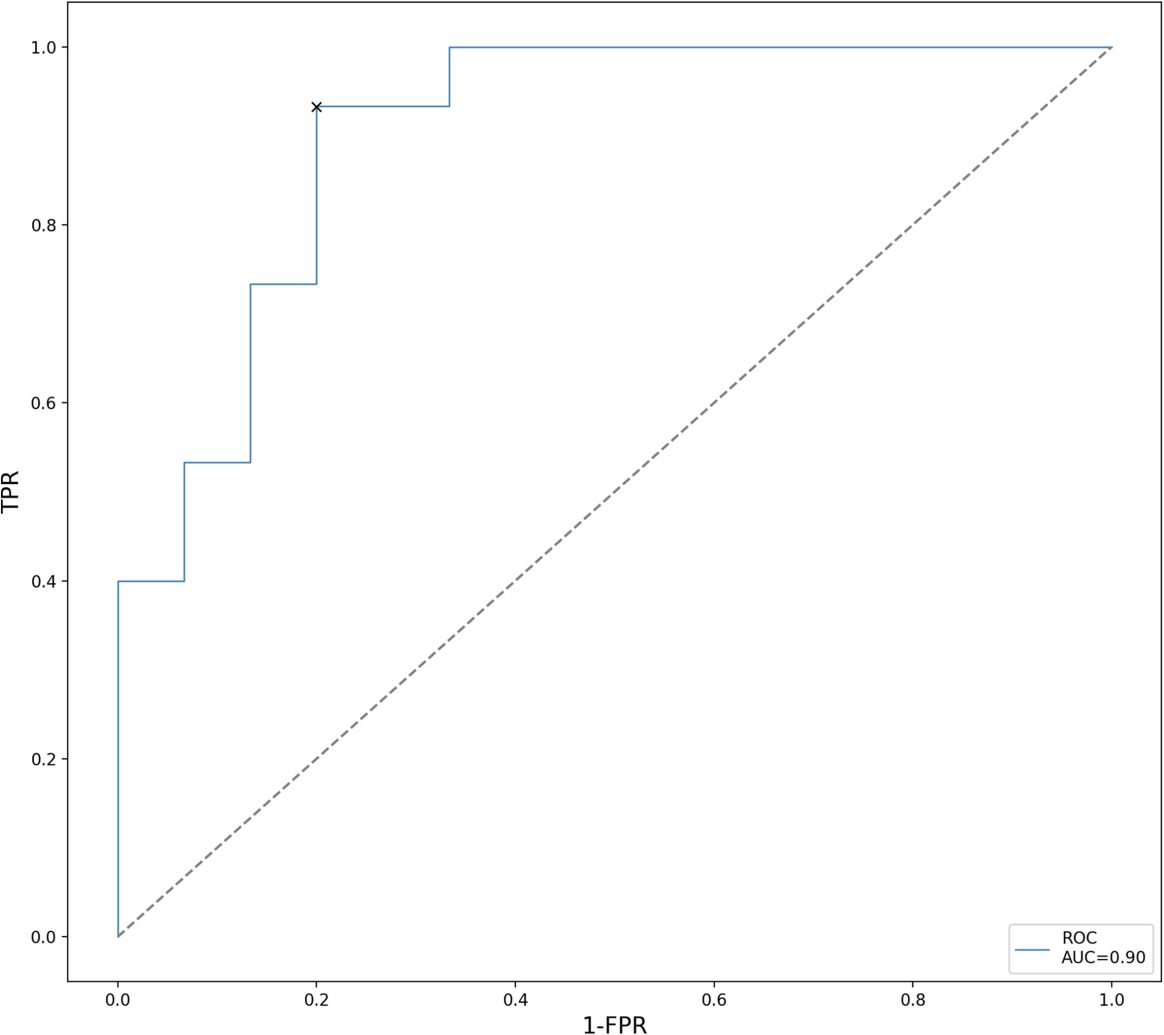
Receiver operator characteristic curve of model performance of the ML algorithm for the independent validation cohort. The classification threshold was 0.5, resulting in an area under the curve of 0.9 (cross) (*n* = 30 patients)

Using cross-validation for algorithm assessment on the training set, the model achieved a sensitivity of 88 ± 13% (mean ± standard deviation), a specificity of 88 ± 10%, and a ROC-AUC of 93 ± 7% (*p* < 0.001, Fisher’s exact test) over the ten cross-validation folds. On the unseen data of the independent validation set, the random forest algorithm achieved a sensitivity of 86.7%, a specificity of 80.0%, a positive predictive value of 81.2%, and a negative predictive value of 85.7%. The area under the ROC curve calculated on the independent validation cohort data was 0.90 (Fig. 2). Results of model evaluation (gain curves and training curves), feature importance metrics, and results from recursive feature elimination as well as Kaplan-Meier modelling of large-area low gray-level emphasis as a singular predictive feature and the results of multivariate Cox analysis and cross-tabulations can be found in Additional file 1.

**Fig. 3:**
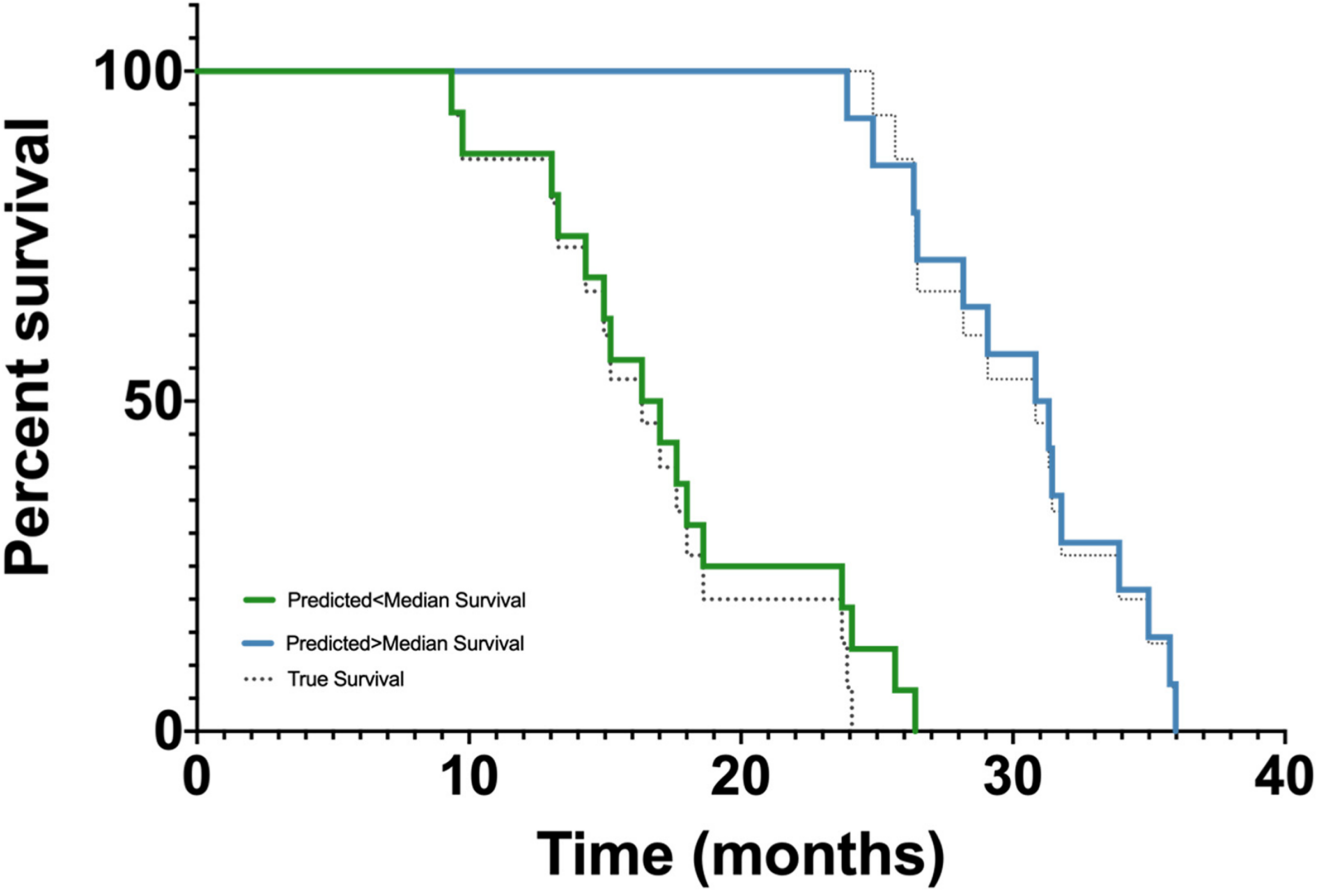
Kaplan-Meier curves showing the predicted survival (blue and green curves) and the true survival (dotted curves) for patients in the independent validation cohort. Log-rank test between predicted survival curves: *p* < 0.001 (*n* = 30 patients)

Furthermore, the algorithm predictions enabled significant stratification of above- *versus* below-median overall survival in the independent validation cohort (*p* ≤ 0.001, log-rank test, predicted median survival for the below-median 17.0 months *versus* 31.3 months for the above-median group) with the resulting predicted survival curves showing near-perfect overlap with the actual survival times of the patients (Fig. 3).

The histopathologic subtype of the tumor samples could be determined for 21 of the 30 patients in the independent validation cohort. The quasi-mesenchymal histopathological subtype was greatly overrepresented in the patient collective predicted by the algorithm to experience below-median survival with 8 out of 9 patients having quasi-mesenchymal subtype tumors. The inverse also held true, with 11 out of 12 patients predicted by the algorithm to experience above-median survival having *non-quasi-mesenchymal* subtype tumors (*p* < 0.001, Fisher’s exact test, Table 2).

**Table 2:** Overlap between predicted survival groups and histopathological subtypes. The quasi-mesenchymal subtype was highly overrepresented in the group with predicted below-median survival, the *non*-*quasi*-*mesenchymal* subtypes in the group with predicted above-median survival (*n* = 30 patients, *p* < 0.001, Fisher’s exact test)

	Quasi-mesenchymal subtype	Non-quasi-mesenchymal subtype
Predicted survival > median	1/12 (9%)	11/12 (91%)
Predicted survival ≤ median	8/9 (89%)	1/9 (11%)

**Fig. 4:**
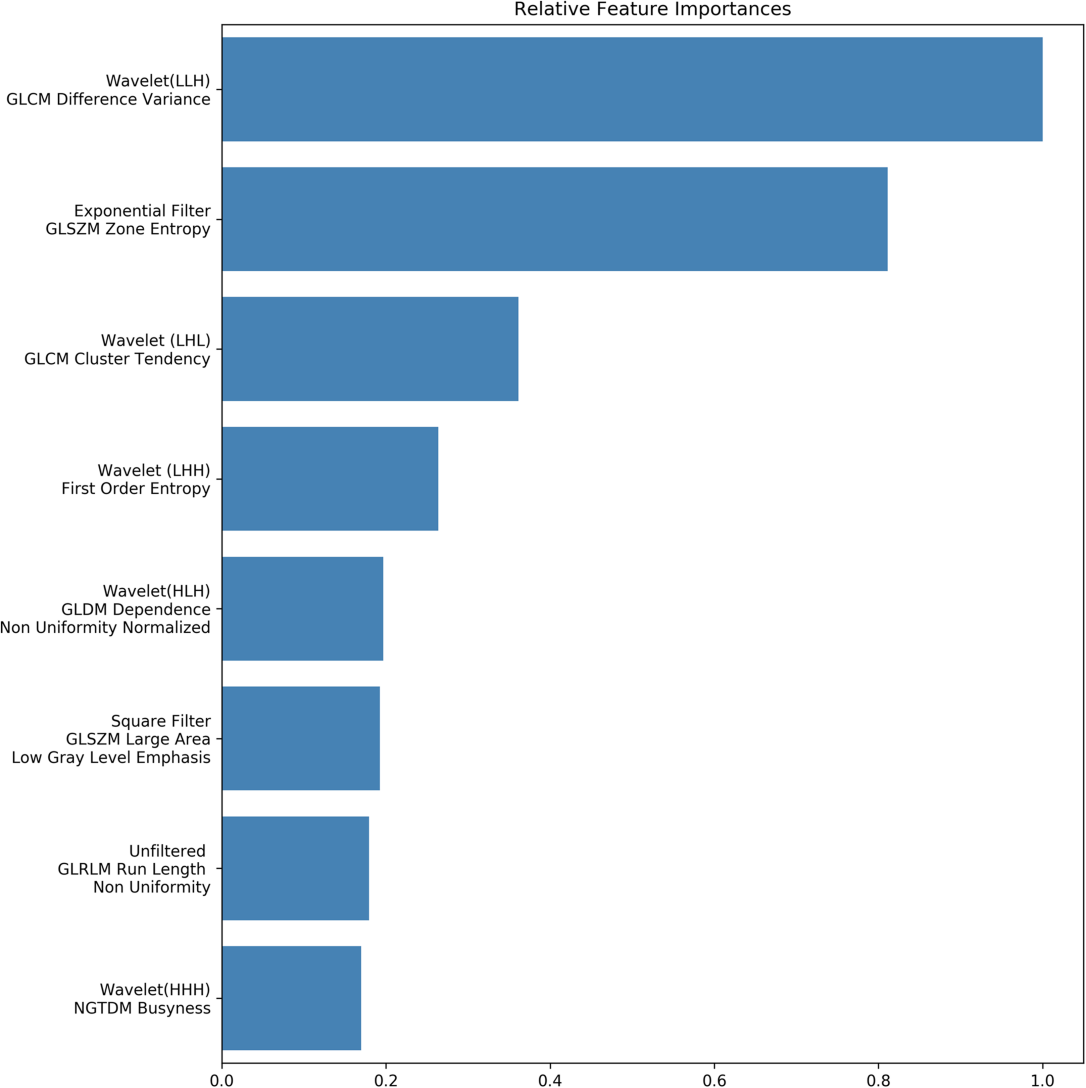
Bar plot of the eight most important features for overall model performance as determined by the random forest model by assessment of Gini impurity decrease and recursive feature elimination. Feature importance has been normalized to the most important feature. The features, in order of descending importance are as follows: (1) gray-level co-occurrence matrix difference variance, (2) gray-level zone size matrix zone entropy, (3) gray-level co-occurrence matrix cluster tendency, (4) first-order entropy, (5) gray-level difference method dependence non-uniformity normalized, (6) gray-level zone size matrix large area low gray-level emphasis, (7) gray-level run-length matrix run-length non-uniformity, and (8) neighbourhood gray tone difference matrix busyness. Of note, features 1–5 are associated with image heterogeneity and only one (6) associated with the proportion of large zones with low gray values within the image

Feature importance evaluation based on node Gini impurity decrease and recursive feature elimination yielded eight highly important features, seven of them associated with image heterogeneity and only one associated with the proportion of large zones with low gray values within the image [12, 13] (Fig. 4).

## Discussion

In this work, we present an independently validated ML algorithm, which enables the prediction of overall survival and shows strong association with histopathologically defined molecular subtypes recently identified in PDAC from preoperative DWI. Several of the most important imaging features belong to a class of *heterogeneity*-*related* features, offering explainable insights into the algorithm.

The potential of radiomics in non-invasive prediction of clinically relevant parameters, such as response to a specific therapy or expected overall survival, has been shown in recent literature: For example, computed tomography-derived radiomic signatures were shown to enable prediction of local disease control and overall survival in PDAC [14, 15] or tumor grading in pancreatic neuroendocrine tumors [16]. The large-scale implementation of such tools thus has the potential to become a *game changer* in medical image interpretation and individualized patient care.

*Post*-*mortem* analyses of terminal stage PDAC specimens have shown higher tumor cellularity compared to resectable PDAC specimens, which likely represent earlier tumor development stages [17]. In line with this observation, we previously demonstrated that higher regional tumor cellularity identified in PDAC resection specimens was associated with a significantly worse overall survival and that the pre-operative DWI-derived ADC parameter could serve as a non-invasive marker thereof [18, 19]. Upholding these findings, the current analysis identified the radiomic feature *large*-*area low gray-level emphasis*, representative of cohesive zones exhibiting low ADC values, as one of the eight most important features for survival classification. Using only this single feature to train the model and predict the survival in the independent validation cohort did, however, not yield statistical significance, with survival curves crossing at early and late time points and only visual separation present at the time interval between 15 and 30 months (see Kaplan-Meier plot and associated metrics in Additional file 1).

Several of the features ranked highly by our model (gray-level co-occurrence matrix difference variance, entropy, non-uniformity, busyness) represent the local heterogeneity of the image. Entropy-related and cluster tendency features were described in the very recent publication by Khalvati et al. [15] as predictive of overall survival in PDAC. Entropy has furthermore been found to represent a highly reproducible and consistent imaging feature in several tumor entities and across modalities [20]. The discovery of such reproducible parameters is a key part of the radiomic process, and it is encouraging to see the same radiomic markers emerge not only across pancreatic cancer studies but also in other tumor entities and across different MRI systems and field strengths, supporting assumptions of overarching ontologies such as tumor heterogeneity and paralleling the notions of pathway—as opposed to tissue-specific therapy approaches [21].

Until proven thoroughly in large prospective trials, the inclusion of ML-derived predictions in a clinical decision process is ethically unjustified. In the future, ML-derived information could be introduced into the clinical work-up of PDAC patients, *e.g*., by back-projection of relevant radiomic features into the image space as it has been demonstrated for prostate cancer [22] and shown in domains outside medical imaging for deep convolutional neural networks [23]. Such visualizations could aid model explainability or offer guidance for invasive tumor sampling in PDAC. The introduction of ML as a clinical decision support tool would also profit from the ability of ML algorithms to predict molecular signatures such as *KRAS* amplification status [24] that may then help stratify patients in clinical routine. Such radiogenomic approaches could complement histomorphology-derived tumor subtype prediction demonstrated here and advance the role of radiomics in precision medicine.

We selected the random forest model over the frequently used linear models such as logistic regression for its capability of modelling both linear and non-linear relationships between features and outcomes, robustness to overfitting by design, and inbuilt insights into feature importance aiding model parsimony and explainability. Random forests have also been shown to yield excellent results in previously published radiomic studies [25].

As part of any radiomic study, feature preprocessing and stability checking is required to obtain reproducible results, resulting in the majority of derived features being discarded before modelling begins [26]. These discarded features are therefore rendered useless for the modelling process. To obtain more usable features, standardized acquisition and feature extraction is necessary. Recent initiatives aim to homogenise acquisition protocols between sites to enable further sequences to be included in analyses [27]. We adhered to (and strongly support) the standards set by the *Image biomarker standardization initiative* and implemented by PyRadiomics [8, 13], which provide a robust post-processing platform entirely based on open-source tools, thus laying the foundation for open and reproducible radiomic science.

Our work is a proof of concept contribution to the fast-developing field of ML in medical imaging. Notable limitations include training cohort size, due to which the model could not reach its full potential performance (see the *training curve* in Additional file 1) and the consensus segmentation approach, which we partially mitigated by excluding features unstable to repeated segmentation in a subcohort of patients. However, Dice-Sørensen overlap scores were not calculated, so no direct data about inter-reader variability is available for the entire cohort, which may limit generalizability. The age of the imaging material in the retrospective training cohort also impacted results with several patients being excluded due to technical image quality. The quality of MRI acquisitions has since considerably improved, and our results could benefit from the application of state-of-the art abdominal imaging including high resolution protocols, such as reduced field-of-view DWI [28–30]. We eliminated all features that were classified unstable between the two MRI systems, and recent research has provided evidence that the quantitative nature of ADC maps results in large numbers of stable features in different tumor entities and across different field strengths and MRI systems [31]. In agreement with these findings, our algorithm maintained high classification performance on independent validation data from a different MRI system, with sensitivity and specificity figures on average 1 to 8% lower than cross-validated performance and with a ROC-AUC reduced by about 3%, indicating that thorough pre-processing, feature engineering and applications using quantitative imaging data can facilitate the deployment of radiomic analyses across systems and institutions.

Further studies on larger cohorts are required to conclusively resolve the impact of switching MRI systems on algorithm generalizability and performance. Lastly, although rigorously quality-controlled, our approach still relies on manual tumor segmentation, since recent fully automated segmentation algorithms fail to match human observers in pancreatic tumors [32]. We believe that future work will result in optimized algorithms that enable a higher level of automation—and thus standardization—of this task.

In conclusion, we showed the promise of ML-based radiomic analyses in PDAC. We encourage the validation of the identified radiomic parameters in larger, prospectively accrued cohorts to lay the foundation for therapeutic interventions based on quantitative imaging biomarkers.

## Additional file

Additional file 1: Supplementary material. (DOCX 403 kb)

## Data Availability

The data generated during the study is available from the corresponding author on reasonable request by a qualified individual or third party.

## Abbreviations

ADC: Apparent diffusion coefficient
AUC: Area under the curve
DWI: Diffusion-weighted imaging
ML: Machine learning
MRI: Magnetic resonance imaging
OS: Overall survival
PDAC: Pancreatic ductal adenocarcinoma
ROC: Receiver operator characteristic

## References

[1] Torphy RJ, Wang Z, True-Yasaki A et al (2018) Stromal content is correlated with tissue site, contrast retention, and survival in pancreatic adenocarcinoma. JCO Precis Oncol:1–12. 10.1200/PO.17.00121 10.1200/PO.17.00121 PMC6262879 30506016

[2] Collisson EA, Sadanandam A, Olson P (2011). Subtypes of pancreatic ductal adenocarcinoma and their differing responses to therapy. Nat Med.

[3] Aung KL, Fischer SE, Denroche RE (2018). Genomics-driven precision medicine for advanced pancreatic cancer: early results from the COMPASS Trial. Clin Cancer Res.

[4] Gillies RJ, Kinahan PE, Hricak H (2016). Radiomics: images are more than pictures, they are data. Radiology.

[5] Aerts HJ, Velazquez ER, Leijenaar RT (2014). Decoding tumor phenotype by noninvasive imaging using a quantitative radiomics approach. Nat Commun.

[6] STROBE Checklist. https://www.strobe-statement.org/index.php?id=available-checklists

[7] ECOG-ACRIN cancer research group. https://ecog-acrin.org/resources/ecog-performance-status.

[8] van Griethuysen JJM, Fedorov A, Parmar C (2017). Computational radiomics system to decode the radiographic phenotype. Cancer Res.

[9] Breiman L (2001). Random forests. Mach Learn.

[10] Hastie T, Tibshirani R, Friedman J (2009). The elements of statistical learning.

[11] Muckenhuber A, Berger AK, Schlitter AM (2018). Pancreatic ductal adenocarcinoma subtyping using the biomarkers hepatocyte nuclear factor-1A and cytokeratin-81 correlates with outcome and treatment response. Clin Cancer Res.

[12] van Griethuysen Joost J.M., Fedorov Andriy, Parmar Chintan, Hosny Ahmed, Aucoin Nicole, Narayan Vivek, Beets-Tan Regina G.H., Fillion-Robin Jean-Christophe, Pieper Steve, Aerts Hugo J.W.L. (2017). Computational Radiomics System to Decode the Radiographic Phenotype. Cancer Research.

[13] Zwanenburg A, Leger S, Vallières M, Löck S (2016) Image biomarker standardisation initiative. CoRR abs/1612.0: 10.17195/candat.2016.08.1

[14] Cozzi L, Comito T, Fogliata A (2019). Computed tomography based radiomic signature as predictive of survival and local control after stereotactic body radiation therapy in pancreatic carcinoma. PLoS One.

[15] Eilaghi A, Baig S, Zhang Y (2017). CT texture features are associated with overall survival in pancreatic ductal adenocarcinoma - a quantitative analysis. BMC Med Imaging.

[16] Liang W, Yang P, Huang R (2019). A combined nomogram model to preoperatively predict histologic grade in pancreatic neuroendocrine tumors. Clin Cancer Res.

[17] Winter JM, Ting AH, Vilardell F (2008). Absence of E-cadherin expression distinguishes noncohesive from cohesive pancreatic cancer. Clin Cancer Res.

[18] Trajkovic-Arsic M, Heid I, Steiger K (2017). Apparent diffusion coefficient (ADC) predicts therapy response in pancreatic ductal adenocarcinoma. Sci Rep.

[19] Heid I, Steiger K, Trajkovic-Arsic M (2017). Co-clinical assessment of tumor cellularity in pancreatic cancer. Clin Cancer Res.

[20] Traverso Alberto, Wee Leonard, Dekker Andre, Gillies Robert (2018). Repeatability and Reproducibility of Radiomic Features: A Systematic Review. International Journal of Radiation Oncology*Biology*Physics.

[21] Schneider G, Schmidt-Supprian M, Rad R, Saur D (2017). Tissue-specific tumorigenesis: context matters. Nat Rev Cancer.

[22] Chatterjee A, Bourne RM, Wang S (2018). Diagnosis of prostate cancer with noninvasive estimation of prostate tissue composition by using hybrid multidimensional MR imaging: a feasibility study. Radiology.

[23] Carter S, Armstrong Z, Schubert L et al (2019) Activation Atlas. Distill 4. 10.23915/distill.00015

[24] Mueller S, Engleitner T, Maresch R (2018). Evolutionary routes and KRAS dosage define pancreatic cancer phenotypes. Nature.

[25] Parmar C, Grossmann P, Rietveld D, Rietbergen MM, Lambin P, Aerts HJ (2015) Radiomic machine-learning classifiers for prognostic biomarkers of head and neck cancer. Front Oncol 5. 10.3389/fonc.2015.00272 10.3389/fonc.2015.00272 PMC4668290 26697407

[26] Kumar V, Gu Y, Basu S (2012). Radiomics: the process and the challenges. Magn Reson Imaging.

[27] Bach M, Röthke M, Henzler T, Kreft M, Amler BSH (2019) Standardized and quality assured prostate diffusion MRI. ECR 2019. 10.26044/ecr2019/C-2163

[28] Ma C, Li Y, Pan C (2014). High resolution diffusion weighted magnetic resonance imaging of the pancreas using reduced field of view single-shot echo-planar imaging at 3 T. Magn Reson Imaging.

[29] Riffel P, Michaely HJ, Morelli JN (2014). Zoomed EPI-DWI of the pancreas using two-dimensional spatially-selective radiofrequency excitation pulses. PLoS One.

[30] Kim H, Lee JM, Yoon JH (2015). Reduced field-of-view diffusion-weighted magnetic resonance imaging of the pancreas: comparison with conventional single-shot echo-planar imaging. Korean J Radiol.

[31] Peerlings J, Woodruff HC, Winfield JM (2019). Stability of radiomics features in apparent diffusion coefficient maps from a multi-centre test-retest trial. Sci Rep.

[32] Zhou Y, Xie L, Shen W, Fishman E, Yuille A (2016) Pancreas segmentation in abdominal CT scan: a coarse-to-fine approach. ArXiv abs/162.08230.

